# Phylogeographic data revealed shallow genetic structure in the kelp *Saccharina japonica* (Laminariales, Phaeophyta)

**DOI:** 10.1186/s12862-015-0517-8

**Published:** 2015-11-02

**Authors:** Jie Zhang, Jian-Ting Yao, Zhong-Min Sun, Gang Fu, Dmitry A. Galanin, Chikako Nagasato, Taizo Motomura, Zi-Min Hu, De-Lin Duan

**Affiliations:** Key Lab of Experimental Marine Biology, Institute of Oceanology, Chinese Academy of Sciences, Qingdao, 266071 China; Qingdao National Laboratory for Marine Science and Technology, Qingdao, 266071 China; University of Chinese Academy of Sciences, Beijing, 100049 China; Sakhalin Scientific Research Institute of Fisheries and Oceanology, Yuzhno-Sakhalinsk, 693023 Russia; Muroran Marine Station, Field Science Center for Northern Biosphere, Hokkaido University, Muroran, 051-0013 Hokkaido Japan

**Keywords:** Demographic history, Genetic diversity, Genetic structure, Gene flow, Mitochondrial DNA, *Saccharina japonica*

## Abstract

**Background:**

Population structure and genetic diversity of marine organisms in the Northwestern Pacific Ocean exhibited complex patterns. *Saccharina japonica* is a commercially and ecologically important kelp species widely distributed along the coast of Japan Sea. However, it is still poorly known about population genetics and phylogeographic patterns of wild *S. japonica* populations on a large geographic scale, which is an important contribution to breeding and conservation of this marine crop.

**Results:**

We collected 612 mitochondrial *COI* and *trn*W-*trn*L sequences. Diversity indices suggested that *S. japonica* populations along the coast of Hokkaido exhibited the highest genetic diversity. Bayesian Analysis of Population Structure (BAPS) revealed four clusters in the kelp species (cluster 1: Hokkaido and South Korea; cluster 2: northwestern Hokkaido; cluster 3: Far Eastern Russia; cluster 4: China). The network inferred from concatenated data exhibited two shallow genealogies corresponding to two BAPS groups (cluster 2 and cluster 3). We did not detect gene flow between the two shallow genealogies, but populations within genealogy have asymmetric gene exchange. Bayesian skyline plots and neutrality tests suggested that *S. japonica* experienced postglacial expansion around 10.45 ka.

**Conclusions:**

The coast of Hokkaido might be the origin and diversification center of *S. japonica*. Gene exchange among *S. japonica* populations could be caused by anthropogenic interference and oceanographic regimes. Postglacial expansions and gene exchange apparently led to more shared haplotypes and less differentiation that in turn led to the present shallow phylogeographical patterns in *S. japonica*.

**Electronic supplementary material:**

The online version of this article (doi:10.1186/s12862-015-0517-8) contains supplementary material, which is available to authorized users.

## Background

The commercially and ecologically important seaweed *Saccharina japonica* (Aresch.) C.E. Lane, C. Mayes, Druehl & G.W. Saunders is widely distributed along the coast of Japan Sea. Taxonomically, this species was initially named *Laminaria japonica* by Areschoug in 1851 [1]. Recently, Lane et al. [2] reported a new genus *Saccharina* Stackhouse from the genus *Laminaria* Lamouroux based on multiple lines of molecular data and proposed to use *S. japonica* to replace *L. japonica*. The systematic survey in Laminariales from the Far Eastern Seas of Russia also supported to transfer *L. japonica* into the genus *Saccharina* [3]. For *S. japonica* along the coast of Hokkaido, integrative morphological and phylogenetic analyses have identified four varieties, *S. religiosa* (Miyabe) C.E. Lane, C. Mayes, Druehl & G.W. Saunders*, S. ochotensis* (Miyabe) C.E. Lane, C. Mayes, Druehl & G.W. Saunders and *S. diabolica* (Miyabe) C.E. Lane, C. Mayes, Druehl & G.W. Saunders [4, 5]. In this study, we did not distinguish these varieties and treated them as one species for phylogeographic analyses.

Global climate change and over exploitation has drastically reduced the resource of *S. japonica* in recent years [6]. Understanding genetic diversity and population structure of wild *S. japonica* populations will aid in the management, conservation and breeding of this marine macroalga. However, current genetic researches of *S. japonica* mainly focused on cultivated populations, and involved wild populations only on a limited geographic scale [7, 8]. The broad picture of wild *S. japonica* populations in the Japan Sea needs clarification, especially regarding population genetic and phylogeographic patterns on a large geographic scale.

**Fig. 1:**
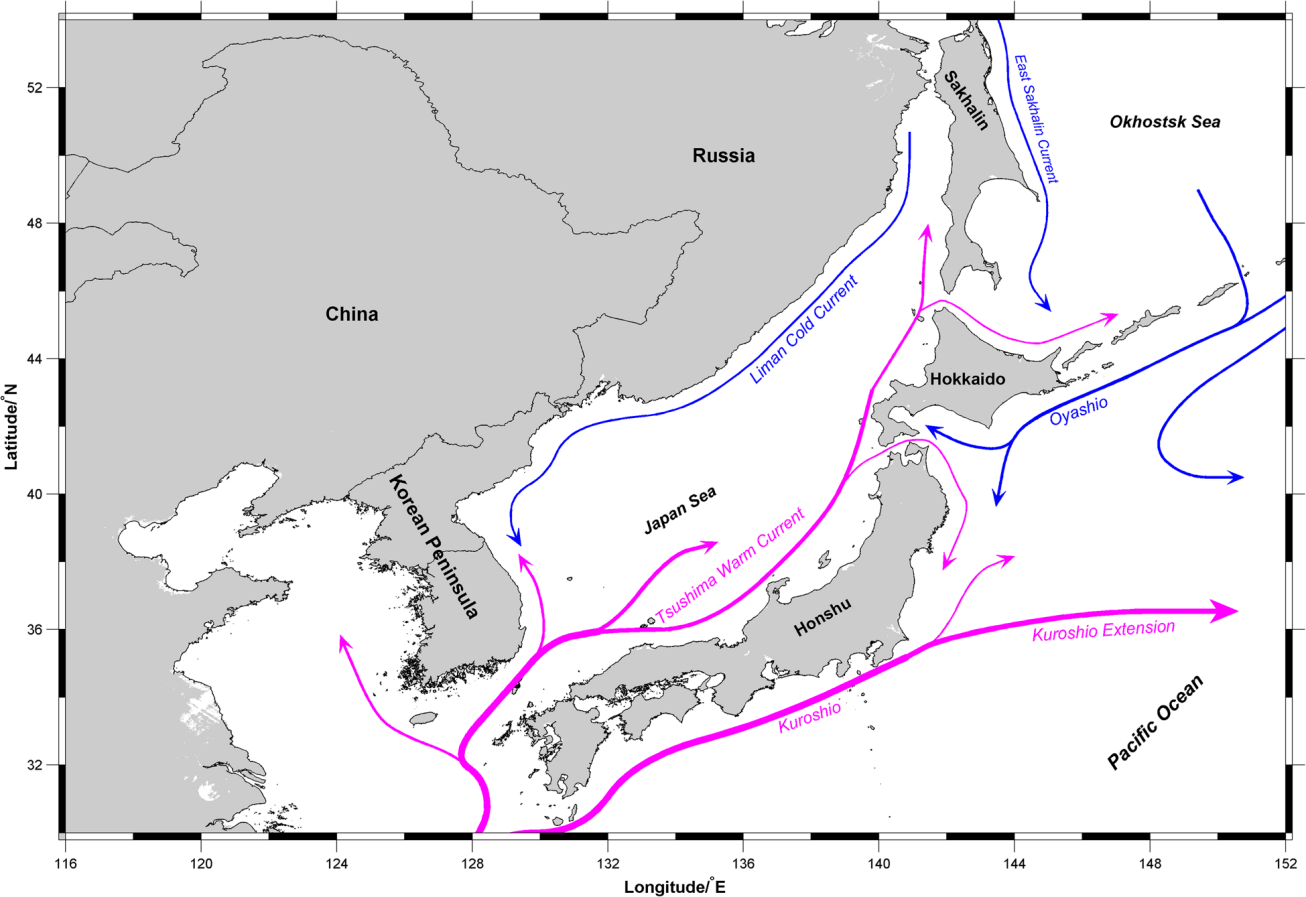
Map showing the sampling locations, and schematic oceanic currents around the Japan Sea. Pink arrows indicate warm currents and blue ones cold currents [14]

Population structure and genetic diversity of marine organisms in the Northwestern Pacific Ocean exhibited complex patterns which mainly resulted from biotic factors (*e.g.* reproductive strategies and intrinsic life-history characteristics) and abiotic factors (*e.g.* complex costal topography, dynamic currents and habitat discontinuities) [9–13]. The semi-isolated marginal Japan Sea is connected with the Sea of Okhostk, the North Pacific, and the East China Sea through four shallow straits of less than 130 m depth [14] (Fig. 1). During the last glacial maximum (LGM), shallow straits restricted or completely blocked inflow of the warm Tsushima current into the Japan Sea via the Tsushima and Tsugaru Straits, reducing sea water temperatures and salinity. After the LGM, higher sea level allowed relatively warm water to flow into the Japan Sea and increased seawater temperature and salinity. Since the mid-Holocene, the modern oceanographic regime of the Japan Sea was established [15–17] and Paleoclimatic oscillations and intricate tectonic topography may have affected the genetic diversity and genetic structure of macroalgae in the Northwestern Pacific [18, 19]. In addition, dispersal processes played an important role in facilitating gene flow in macroalgal populations and structuring the established populations [20–23]. Along with short-lived spores and gametes, macroalgae have relatively poor dispersal ability [24]. However, ocean currents facilitated dispersal over long distances for organisms such as *Laminaria digitata* (Hudson) J.V. Lamouroux and *Macrocystis pyrifera* (Linnaeus) C. Agardh [21, 22]. Incident anthropogenic introduction of *S. japonica* influenced dispersal and gene flow of brown seaweed, especially economic seaweeds [7, 8, 25]. In the wild environment, which kinds of factors affected the genetic structure and phylogeographic pattern of *S. japonica* are rarely known.

Mitochondrial *COI* and *trn* DNA markers have been intensively used to explore intra-specific phylogeographic patterns of brown seaweeds [18, 26–32], including the kelp *S. japonica* [5]. In particular, *COI* marker has been demonstrated to has more polymorphism sites than nuclear (ITS) and plastid (*rbc*LS), and has been verified in *S. japonica* [33]. In the present study, we chose mitochondrial *COI* and *trn*W-L and applied them in 26 *S. japonica* wild populations to explore intraspecific genetic diversity, population structure and demographic history and to find potential abiotic and/or biotic factors associated with genetic differentiation in *S. japonica*.

## Methods

### Sample collection

**Fig. 2:**
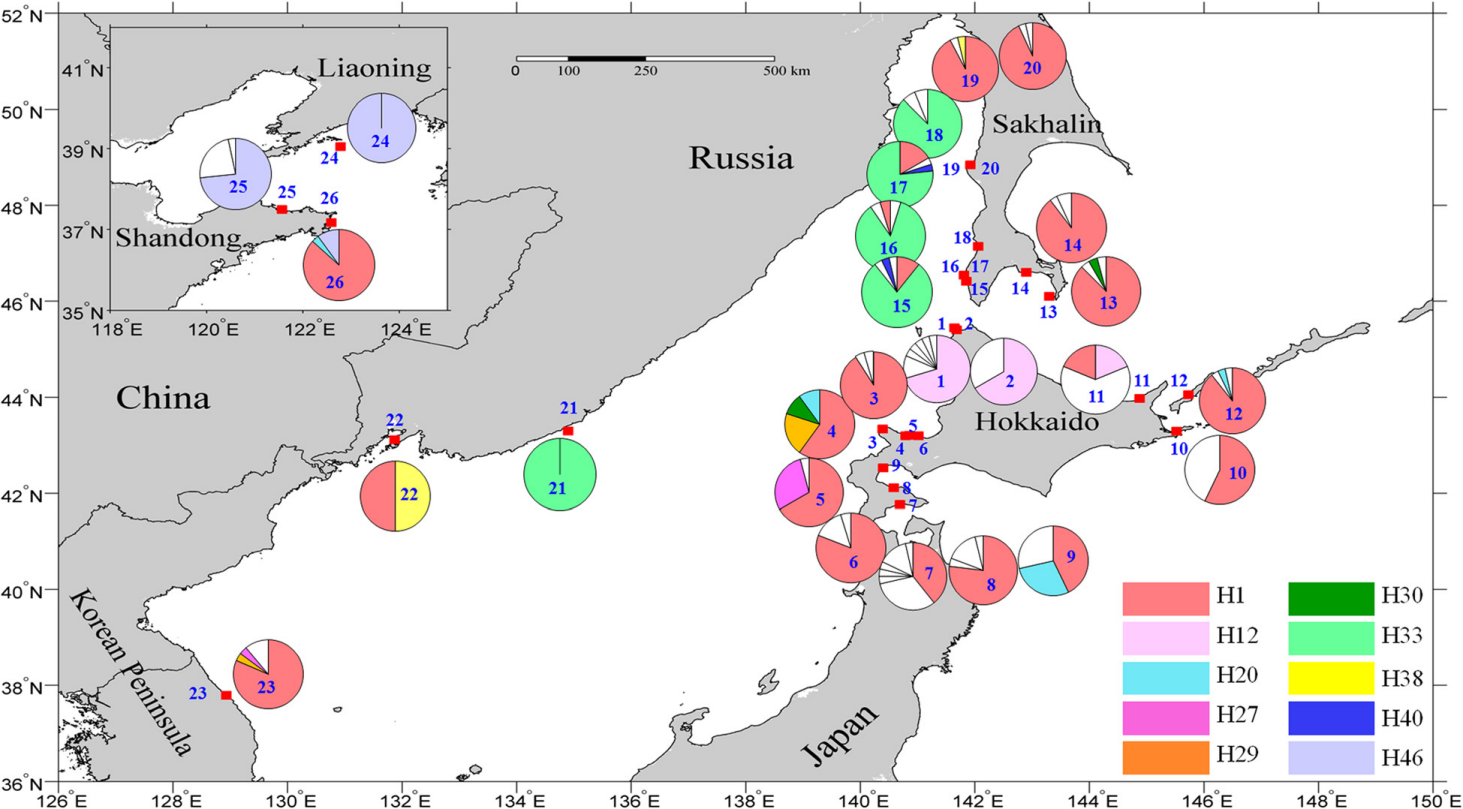
Distribution of the 53 combined mtDNA haplotypes detected in the 26 *Saccharina japonica* populations. Pie charts are labeled with the population numbers as shown in Table 1. The size of divisions inside each pie chart is proportional to the frequency of occurrence for individual haplotypes. Colors represent the shared haplotypes and private haplotypes are shown in white

Between 2011 and 2013, 26 populations of *S. japonica* (612 individuals) were collected, ranging from Sakhalin, Russia (48°50′ N) to Shandong, China (37°09′ N) (Fig. 2; Additional file 1: Table S1). The collection covered most kelp distribution locations along the coast of the Northwestern Pacific Ocean.

### DNA extraction, PCR and sequencing

*S. japonica* genomic DNA was extracted using the Plant genomic DNA kit (Tiangen, Beijing) according to manufacturer’s instructions and mitochondrial markers *COI* and *trn*W-L were adopted. One *COI* partial region was amplified with primers SacCOIF and SacCOIR [33] and the *trn*W-L partial region from the 3′ end of the *trn*W gene to the 5′end of the *trn*L was amplified with primers 5′-GGTTCAAGTCCCTCTCTTTCTGT-3′ (*trn*W-LBF, forward) and 5′-AACCTAAACCCAGCGTGTAT-3′ (*trn*W-LBR, reverse) based on the mitochondrial genome sequence of *S. japonica* [5]. The PCR mixture (50 μL) containing 50 ng genomic DNA, 0.2 mM dNTP, 1 × *Taq* buffer, 0.4 μM of each primer, 0.5 unit *Taq* polymerase and sterile water (Transgen, Beijing) was amplified on a Takara thermocycler (Takara Bio, Japan) with initial denaturation of 95 °C for 5 min, 35 cycles of 95 °C denaturation for 30 s, 52 °C (*COI*)/55 °C (*trn*W-L) annealing for 30 s, a 72 °C extension for 2 min (*COI*) or 1 min (*trn*W-L) and final extension of 72 °C for 5 min. Amplified products were sequenced on an ABI PRISIM 3730 automatic sequencer (Applied Biosystems, USA) and sequenced data were edited and aligned with BioEdit v7.1 software [34].

### Data analysis

#### Genetic diversity and haplotype network construction

The number of segregating sites (S), average number of nucleotide differences (K), nucleotide diversity (Pi) and haplotype diversity (Hd) were calculated using DNASP v5 [35]. NETWORK v4.5.1.6 [36] was used to generate the maximum parsimony media-joining network, which exhibited genealogical relation to the haplotypes of *S. japonica.* To test mutation rate, *COI* and *trn*W-L sequence data were concatenated and the combined mtDNA haplotype network was constructed.

### Population structure and gene flow

With concatenated data of two mtDNA loci, Bayesian analysis of population structure was conducted using BAPS v6.0 with a spatial model to define groups [37, 38]. Calculations were performed with the upper number of K clusters varying from 2 to 26, providing the highest posterior probability for reasonable partitioning. Hierarchical molecular variance analysis (AMOVA) was performed on the four BAPS groups with 10,000 permutations using Arlequin v3.5 [39]. Estimated *F*_ST_ values of pairwise populations and pairwise BAPS groups were conducted for genetic differentiation analysis using 10,000 permutations with Arlequin v3.5.

The mantel test for isolation-by-distance (IBD) established relationships between genetic and geographic distances [40] and genetic distances (*F*_ST*/*_(1*–F*_ST_)) were regressed against the logarithm of geographic distances within location distances (estimated with Google Earth 6.0). To visualize IBD patterns [41], 1000 randomizations were analyzed with IBDWS (http://ibdws.sdsu.edu/~ibdws/).

To further test migration rate and divergence time between neighboring populations in Far Eastern Russia and Hokkaido, an isolation-with-migration model was applied using software IMa2 [42, 43]. Migration rate for population pairs in IMa2 had to be estimated due to computational challenges related to the high number of parameters from multiple populations. Preliminary runs were performed to optimize upper bounds on prior distributions (q = 10, t = 10, m = 10; where q = population size, t = divergence time, and m = migration rate) and to optimize heating schemes. Final analyses consisted of three runs of 10–60 geometrically heated chains with burn-in of 500,000 steps. The heating scheme used a geometric model with parameters ha = 0.96 and hb = 0.9. A total of 120,000 genealogies were saved after the three long runs and used to calculate parameter values and likelihood ratio tests of nested speciation models [43].

### Molecular clock calibration

According to the study of Silberfeld et al. [44] on brown algae, the divergence time of *Saccharina* occurred approximately 20 Ma ago. Therefore, 626 bp *COI* was used to re-construct the phylogeny of Laminariales (3 genera and 5 species) and 20 Ma was used as the ancestral time of the common ancestor (*t*_*MRCA*_) of *Saccharina, Pelagophycus* Areschoug and *Nereocystis* Postels & Ruprecht, which all are implemented in BEAST v1.7.4 [45] with the uncorrelated log-normal (UCLN) relaxed-clock model and the HKY model based on the Bayesian information criterion analysis in PartitionFinder v1.1.0 [46]. The maximum clade credibility tree was constructed with TreeAnnotator v1.7.5 [47].

### Demographic history

The duration of transplantation and domestication to *S. japonica* in China and Korea is short compared with terrestrial crops, so neutrality tests and mismatch distribution analysis were used to explore the demographic history of the 22 *S. japonica* populations (representing all populations examined in this study except for four populations in China and Korea. Tajima’s *D* [48] and Fu’s *F*_*S*_ [49] were estimated and a mismatch distribution test was administered with reference to the method of Schneider et al. [50]. A Bayesian skyline plot (BSP) was applied for the historical demographic analysis of *S. japonica* using BEAST v1.7.4 [51]. *COI* or *trn*W-L marker and concatenated data (*COI* and *trn*W-L) was used for data analysis, with mean substitution/site selected at 1.0 for the time estimation to the units of substitution/site. The HKY + I substitution model was selected for *COI*, the HKY substitution model was applied for *trn*W-L and the HKY + G substitution model was selected for combined sequences as identified in PartitionFinder v1.1.0 [46]. The number of grouped intervals was set at 15, and each run was initiated with random starting trees having chain lengths of 3 × 10^7^ and 10^8^. For each marker, multiple analyses were conducted with different random seeds to test convergence, and results from replicate runs were pooled with LogCombiner v2.1.2 and parameters were assessed using Tracer v1.4.1 [51].

## Results

### Haplotype patterns and network

With alignment and trimming, the remaining sequences were 1528 bp for *COI* and 362 bp for *trn*W-L. In the *COI* and *trn*W-L sequences from 612 individuals, 35 and 20 segregating sites respectively were identified, possessing 30 *COI* haplotypes (GenBank accession number: KT963115-KT963144) and 22 *trn*W-L haplotypes (GenBank accession number: KT963093-KT963114).

Calculated genetic variations are summarized in Table 1. For the *COI* marker, nucleotide diversity (Pi) range was 0.00000–0.00191 and haplotype diversity (Hd) range was 0.0000–0.68254. For the *trn*W-L marker, haplotype diversity was 0.00000–0.0042 and nucleotide diversity was 0.00000–0.76190. Data from both *COI* and *trn*W-L showed highest diversity indices in Hokkaido, Japan. For the whole dataset (*COI* + *trn*W-L sequences, 1890 bp), there were 55 segregating sites and 53 haplotypes in all the 26 populations (Table 1). Haplotype diversity (Hd) of total populations was 0.72165, with a range of 0.0000–0.76190 (Table 1), and haplotype diversity in Hokkaido (Hd = 0.72528) was higher than in other populations (Russia 0.58537; Korea 0.03333; China 0.55306) (Table 1). Highest nucleotide diversity was also detected in Hokkaido populations (0.00079) (Table 1). Generally, diversity parameters yielded from the two combined mtDNA markers indicated that pop7 and pop9 possessed the highest nucleotide and haplotype diversity and pop21 (Russia) and pop24 (China) exhibited the lowest nucleotide and haplotype diversity (Table 1). All genetic diversity parameters indicated that the *S. japonica* populations along the coast of Hokkaido exhibited the highest genetic diversity.

**Table 1:** The summary of genetic diversity in *Saccharina japonica*

POP	N	*COI*				*trn*W-L				*COI* + *trn*W-L			
		S	Pi	Hd	h	S	Pi	Hd	h	S	Pi	Hd	h
**Japan**	**231**	**19**	**0.00067**	**0.47149**	**15**	**16**	**0.00131**	**0.39029**	**16**	**35**	**0.00079**	**0.72528**	**30**
1	27	5	0.00041	0.44160	5	3	0.00113	0.33333	4	8	0.00055	0.50427	7
2	6	1	0.00035	0.53333	2	0	0.00000	0.00000	1	1	0.00028	0.53333	2
3	22	0	0.00000	0.00000	1	2	0.00050	0.17749	3	2	0.00010	0.17749	3
4	10	2	0.00047	0.35556	2	2	0.00110	0.37778	3	4	0.00059	0.64444	4
5	24	2	0.00056	0.43116	2	1	0.00023	0.08333	2	3	0.00050	0.48913	3
6	21	1	0.00006	0.09524	2	1	0.00071	0.25714	2	2	0.00019	0.33810	3
7	28	8	0.00191	0.68254	5	1	0.00039	0.14021	3	10	0.00162	0.74339	7
8	26	2	0.00026	0.38462	3	1	0.00021	0.07692	2	3	0.00025	0.39692	4
9	7	0	0.00000	0.00000	1	3	0.00421	0.76190	3	3	0.00081	0.76190	3
10	28	0	0.00000	0.00000	1	1	0.00140	0.50794	2	1	0.00027	0.50794	2
11	32	1	0.00021	0.31452	2	1	0.00134	0.48387	2	2	0.00042	0.55645	3
**Russia**	**267**	**16**	**0.00009**	**0.10935**	**14**	**9**	**0.00159**	**0.52984**	**9**	**25**	**0.00038**	**0.58537**	**22**
12	28	1	0.00005	0.07143	2	2	0.00039	0.14021	3	3	0.00011	0.20635	4
13	24	2	0.00011	0.16304	3	1	0.00023	0.08333	2	3	0.00013	0.23913	4
14	29	2	0.00013	0.19704	3	0	0.00000	0.00000	1	2	0.00011	0.19704	3
15	28	2	0.00009	0.14021	3	2	0.00090	0.31481	3	4	0.00025	0.38095	5
16	21	2	0.00012	0.18571	3	1	0.00026	0.09524	2	3	0.00015	0.27143	4
17	30	1	0.00004	0.06667	2	3	0.00139	0.38391	3	4	0.00030	0.39540	4
18	16	1	0.00008	0.12500	2	1	0.00035	0.12500	2	2	0.00013	0.24167	3
19	26	1	0.00005	0.07692	2	2	0.00042	0.07692	2	3	0.00012	0.15077	3
20	29	5	0.00023	0.13547	3	0	0.00000	0.00000	1	5	0.00018	0.13547	3
21	30	0	0.00000	0.00000	1	0	0.00000	0.00000	1	0	0.00000	0.00000	1
22	6	0	0.00000	0.00000	1	2	0.00331	0.60000	2	2	0.00063	0.60000	2
**Korea**	**27**	**5**	**0.00033**	**0.33333**	**4**	**0**	**0.00000**	**0.00000**	**1**	**5**	**0.00027**	**0.03333**	**4**
23	27	5	0.00033	0.33333	4	0	0.00000	0.00000	1	5	0.00027	0.03333	4
**China**	**87**	**1**	**0.00028**	**0.43304**	**2**	**3**	**0.00146**	**0.49051**	**3**	**4**	**0.00051**	**0.55306**	**5**
24	27	0	0.00000	0.00000	1	0	0.00000	0.00000	1	0	0.00000	0.00000	1
25	30	0	0.00000	0.00000	1	3	0.00149	0.42069	3	3	0.00028	0.42069	3
26	30	1	0.00012	0.18621	2	1	0.00066	0.23908	2	2	0.00023	0.24598	3
Total	612	35	0.00043	0.40824	30	20	0.00191	0.56200	22	55	0.00072	0.72165	53

*N* number of sequences, *S* number of segregating sites, *Pi* nucleotide diversity, *Hd* haplotype diversity, *h* number of haplotypes in each population. The bold indicated that the summary genetic diversity of all populations in this regions

**Fig. 3:**
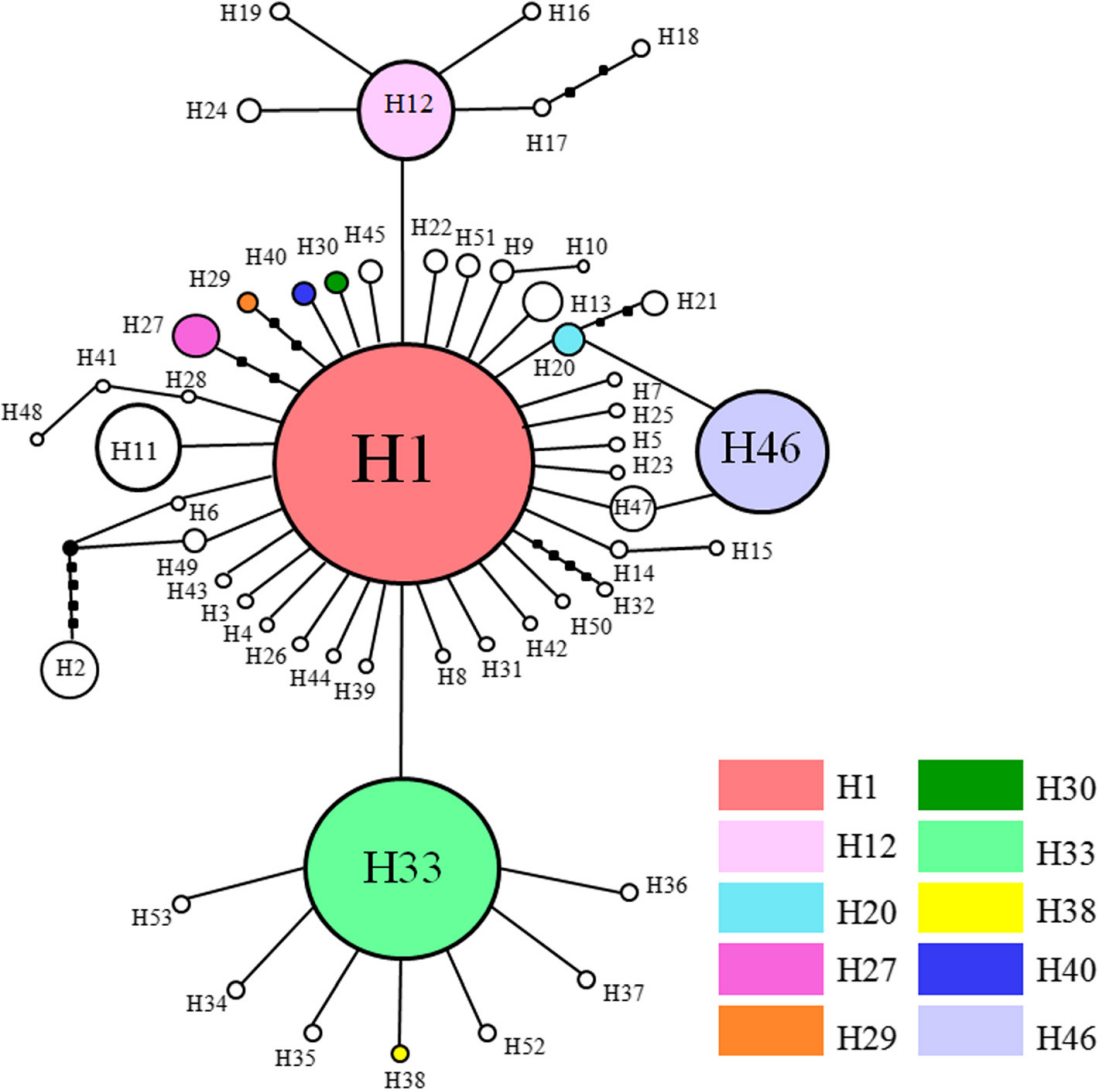
Median-joining network constructed using the haplotypes of the concatenated sequences. Circle size is proportional to sample size for each population (*n* = 1-298 individuals). Missing haplotypes are shown as dots in the network. Each connecting line indicates one mutation step between haplotypes and each black dot represents one mutation step. Colors represent shared haplotypes and private haplotypes are shown in white

There were 10 shared haplotypes and 43 private haplotypes, which were unique to a single population based on combined sequences (Fig. 2). The most frequent and widespread haplotype H1 was found in all geographic regions and the main haplotype H33 lay in Far Eastern Russia. H20 was shared by Hokkaido pop 4, pop9, pop12 and pop26. H30 was shared by Hokkaido pop 4 and Sakhalin pop 13. H1 was in 48.69 % of individuals and 76.92 % of populations. Moreover, 30 haplotypes were definable with a single mutation difference from H1, with H2 seven mutation steps removed from H1 (Fig. 3).

### Genetic structure and gene flow

Bayesian Analysis of Population Structure (BAPS) revealed four groups (Additional file 2: Figure S1). Cluster 1 contained 14 populations not otherwise partitioned. Cluster 2 included pop1, pop2, pop7 and pop11 in Hokkaido, while cluster 3 contained 6 populations, which located in Far Eastern Russia and cluster 4 contained 2 wild populations (pop24 and pop25) in northern China. AMOVA tests indicated significant differentiation among all populations (*F*_CT_ = 0.546, *P* < 0.001) defined by BAPS, which accounted for 54.79 % of variation (Table 2). Pairwise *F*_ST_ values among the four clusters ranged from 0.22309 (cluster 1 and cluster 4) to 0.87016 (cluster 2 and cluster 3) (Additional file 3: Table S2). Pairwise *F*_ST_ tests exhibited low or moderate genetic differentiation (Additional file 4: Table S3). Overall, pop1 and pop2 in Hokkaido have deep divergence with other populations (*F*_ST_: 0.384–0.9336). Pairwise *F*_ST_ values indicated that pop24 and pop25 in China diverged significantly from the other 24 populations (*F*_ST_: 0.5304–1.000) (Additional file 4: Table S3).

**Table 2:** Analysis of molecular variance based on pairwise differences of *COI* and *trn*W-L combined sequences. The analysis was run independently using populations grouped by BAPS groups

Source of variations	Degree of freedom	Variance components	Percent of variance	Fixation indices
Among Groups	3	0.46766	54.79	*F*_CT_ =0.54786^**^
Among populations within Groups	22	0.10206	11.96	*F*_SC_ *=*0.26443^**^
Within populations	586	0.28389	33.26	*F*_ST_ = 0.66742^**^

**P< 0.001; *P< 0.05

The mantel test indicated no significant correlation between genetic distance (*F*_ST_/(1–*F*_ST_)) and geographical distance (22 populations except populations in China and Korea) (Additional file 5: Figure S2). Regression coefficients (R^2^ < 0.01) indicated that the population genetic structure of *S. japonica* did not fit the IBD model.

**Fig. 4:**
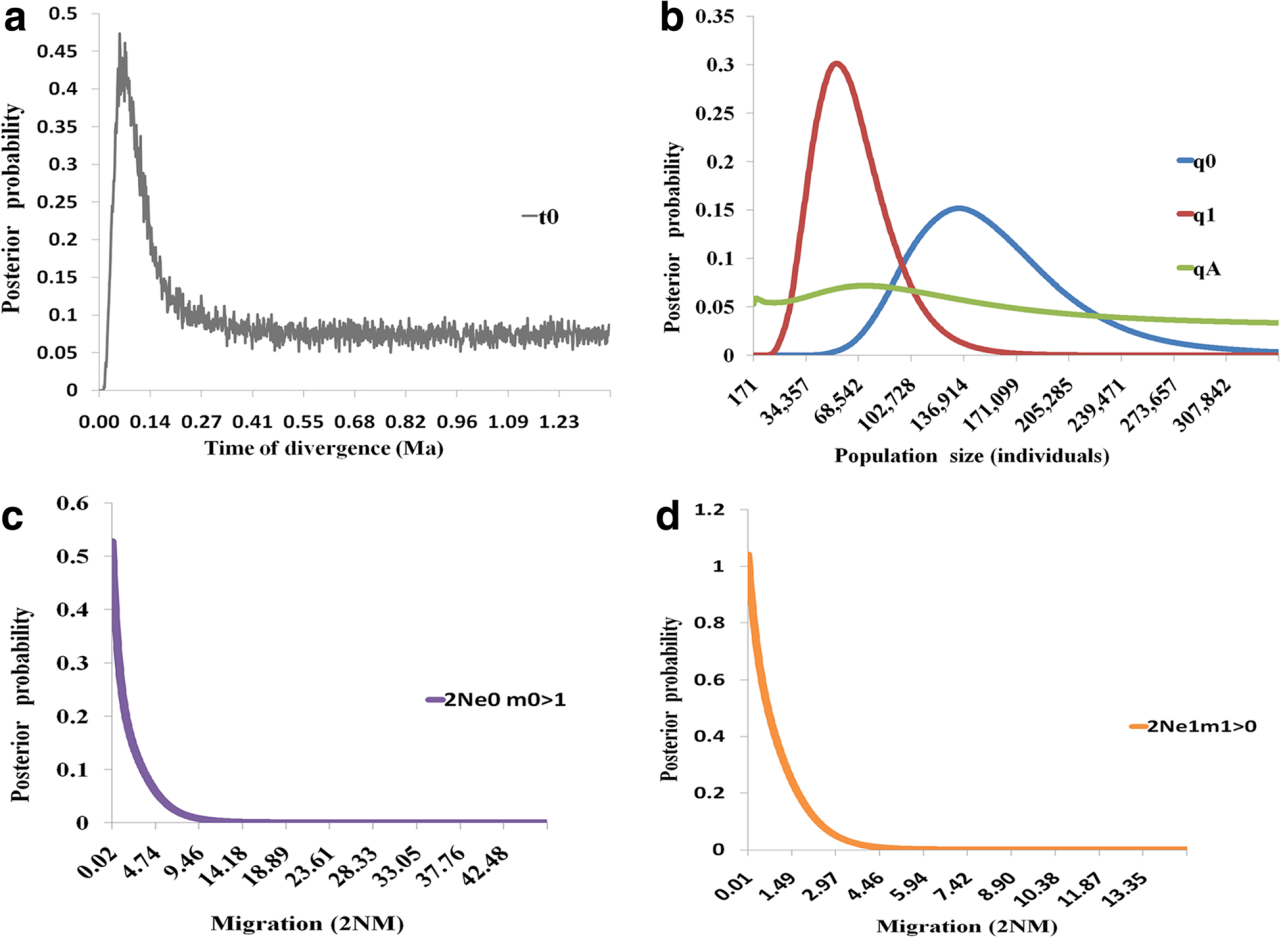
Results of pairwise IMa analysis of *Saccharina japonica* populations based on combined mtDNA sequences. **a** Posterior densities for splitting time (t0); (**b**) Posterior densities for population sizes (q0, q1, qA); (**c, d**) Posterior densities for population migration (2N0m0 > 1 and 2N1m1 > 0)

Posterior probability distributions, peak posterior point estimates and 95 % confidence intervals of parameters are in Table 3. Two lineages (cluster 2 and cluster 3) had clear geographic distribution and were divided into 5 neighboring units: Validivostok (VL, pop21 and pop22), west coast of Sakahin (WS, pop15–18), Wakkanai (WA, pop1 and pop2), Shari (SH, pop12) and Hakodate (HA, pop7). IMa2 was ran for three pairs of populations in *S. japonica* (pair 1: WS versus VL; pair 2: WA versus SH; pair 3: WA, HA & SH versus VL & WS) reaching convergence with high ESS values across all parameters. Migration between cluster 2 and cluster 3 (pair 3) seemed to be negligible in both directions (HiPt: 0.02359, 95 % HPD: 0.00–7.147; in the opposite direction, HiPt: 0.007412, 95 % HPD: 0.00–2.780). Effective population sizes (HiPt: 1.3 × 10^5^, 95 % HPD: 6.8 × 10^4^–2.6 × 10^5^) were approximately two times higher in cluster 2 than in cluster 3 (HiPt: 5.4 × 10^5^, 95 % HPD: 2.2 × 10^4^–1.1 × 10^5^). Peak posterior distribution of migration rate estimated asymmetrical gene flow between WA and SH, where migration to SH was significant (HiPt: 44.98, 95 % HPD: 0.0–2822) and migration to WA was negligible (HiPt: 0.3907, 95 % HPD: 0.0–2.030). A similar asymmetrical gene flow was observed between VL and WS, with gene flow from VL to WS was significantly higher than in the opposite direction. Estimated split time between cluster 2 and cluster 3 was 0.0554 Ma (HiPt: 0.0554, 95 % HPD 0.0226–1.3667), with all statistics sharing broadly overlapping 95 % HPD intervals (Table 3, Fig. 4a).

**Table 3:** Estimates of populations sizes, migration rates and effective number of migrants for pairwise population comparisons. Upper and lower confidence limits of 95 % highest posterior probability intervals are given for each parameter. Demographic estimates were calculated using *COI* sequence mutation rate of 7.313 × 10^−6^ mutations per site per year. Population migration rates, 2Ne_0_ m_0>1_ and 2Ne_1_ m_1>0_ are scaled by effective population sizes, where 2Ne_0_ m_0>1_ is the effective number of migrants into population 1 from population 0 and 2Ne_1_ m_1>0_ is the effective number of migrants into population 0 from population 1. q_0_, q_1_ and q_A_ are the estimates of population size for populations 0, population 1 and ancestral population, respectively

	q_0_	q_1_	q_A_	t_0_ (Ma)	2Ne_0_ m_0>1_	2Ne_1_ m_1>0_
Pair 1 (WS versus VL): 0 = pop21&pop22; 1 = pop15,pop16,pop17&pop18						
HiPt	136674	15384	683.7	0.0001	0.8996	22.49
HPD95Lo	269338	41775	0.0	0.0	0.0	0.0
HPD95Hi	15515528	136674	10250581	1123342	378.1	25255
Pair 2 (WA versus SH): 0 = pop1&pop2; 1 = pop12						
HiPt	263230	1308	37604	0.6033	44.98	0.3907
HPD95Lo	71790	282.0	0.0	0.1132	0.0	0.0
HPD95Hi	6327772	9051	1297005	1.0934	2822	2.030
Pair 3 (WA, HA & SH versus VL & WS): 0 = pop1,pop2,pop7&pop12; 1 = pop15-18,pop21&pop22						
HiPt	133837	54184	72645	0.0554	0.02359	0.007412
HPD95Lo	68200	22050	0.0	0.0226	0.0	0.0
HPD95Hi	259299	115719	316047	1.3667	7.147	2.780

### Demographic history

The significantly negative Tajima’s *D* and Fu *F*_*S*_ values indicated that *S. japonica* populations might experience demographic expansion (Additional file 6: Figure S3). Using a single *COI* marker or *trn* W-L marker for mismatch distribution, displayed L-shaped and reflected shallow haplotype phylogeny in *S. japonica* populations (Additional file 6: Figure S3a and b). Moreover, the mismatch distribution of combined haplotypes showed a unimodal pattern, indicating that *S. japonica* populations might have expanded. In addition, Bayesian skyline plots analyses showed that *S. japonica* population underwent population expansion (Additional file 7: Figure S4).

With a maximum clade credibility tree based on the *COI* data (Additional file 8: Figure S5), divergent time between the *S. japonica* and *S. augustata* (Kjellman) C.E. Lane, C. Mayes, Druehl & G.W. Saunders was assumed to be 4.84 Ma. Calculated *COI* region divergence between *S. japonica* (AP011493) and *S. augustata* (AP011498) was 4.633 % and estimated divergent rate was 0.9572 %/Ma (4.633 %/4.84 Ma, rounded conservatively) [52]. Since mutation rate should be half of divergence rate [53, 54], estimated mutation rate was 0.4786 %/Ma. Bayesian skyline plots and the calculated molecular clock of *COI* suggested that *S. japonica* populations started to expand at 10.45 Ka (Additional file 7: Figure S4a).

## Discussion

### Two shallow genealogies existed in *S. japonica* populations

Data sets revealed a shallow genetic structure in *S. japonica*, with many shared haplotypes in four genetic clusters (Fig. 2; Additional file 2: Figure S1). The combined network does not have large divergences between haplotype groups and two shallow genealogies corresponded to two BAPS groups (cluster 2 and cluster 3) (Fig. 3; Additional file 2: Figure S1). Most kelp populations in these two genealogies were isolated by the Soya Strait, a major outlet of the Tsushima Warm Current [55]. The current flow direction has hindered dispersal of kelp, producing low levels of gene flow (2 Nm < 1) in both directions between these two genealogies (Table 3; Fig. 4c and d). The Tsushima Warm Current flows through the Soya Strait and enhanced the sea water temperature and salinity [55], so the northern coast of Hokkaido has higher sea temperature and lower salinity than the west coast of Sakhalin, which might lead to different release time of spores, different discharge time of sperm and different egg fertilization time [56, 57]. Distinct marine environmental factors and non-simultaneous reproductive duration likely caused these two genealogies isolation and influenced the formation of them.

### Postglacial expansion, oceanic current and anthropogenic introduction influence the current genetic structure

Mismatch distributions, neutrality tests and BSP analyses all suggested that *S. japonica* expanded in the Northwestern Pacific Ocean and calculations using the *COI* molecular clock suggested that such population expansion occurred about 10.45 Ka. Post-LGM expansion (25–15 Ka) significantly influenced the demography of seaweeds [28, 60]. The paleoceanographic record in the Japan Sea shows that sea water temperature and salinity were lower (drop 140 m of sea level) during the LGM (25–15 Ka) and seawater exchange through straits was blocked. After the LGM, sea water temperature rose in the early Holocene (8–10 ka) due to the inflow of Tsushima Warm Current and sea temperature rose 6–7 °C. Meanwhile, the salinity of seawater in the Japan Sea increased during the interglacial period. With the cold-adapted and lower salinity tolerant characters, *S. japonica* could survive severe ancient conditions and colonized suitable coastal territories when the sea level increased after the LGM. Judging from the constructed haplotype network (Fig. 3) and IMa analyses (Fig. 4 and Table 3), pink and green genealogies had no contemporary gene flow since they diverged in mid-Pleistocene, showing that these two genealogies might be existed at least in two glacial refugia in the Japan Sea coast during LGM and afterwards, expanded their populations to the current distribution in the interglacial period.

Low genetic differentiation in the populations of *S. japonica* suggested gene exchange among detected populations. In addition, there were several shared haplotypes and no significant population differentiation corresponding to geographic distribution (Fig. 3; Additional file 2: Figure S1), indicating gene flow in kelp populations. Shared haplotype H30 and H20 indicated that some populations in Sakhalin and Hokkaido had gene exchange. Lower gene flow existed in brown algae such as *Fucus ceranoides* Linnaeus and *Laminaria digitata* (Hudson) Lamouroux [22, 58] due to factors such as short-lived spores and gametes and reproductive strategies [20, 22, 24]. Macroalgae are species with relatively poor dispersal ability, but *S. japonica* in the present study has high gene flow among most populations. In *S. japonica,* due to limited gametes and spores dispersal ability, the floating or drifting thallus by oceanic currents was regarded as the main dispersal factor to enhance the gene flow in the kelp populations [8, 57]. The warm Tsushima Current, flows northward along the western coast of Hokkaido, and enters into Sea of Okhostk through the Soya Strait, then forwards along coast of northern Hokkaido. The Tsushima current is likely the major force responsible for drifting kelp thallus from the eastern coast of Hokkaido to Kunashir Island, evidenced by asymmetric gene flow between WA and SH (Table 3).

Marine transportation also promotes kelp population genetic homogeneity. Pop 21 (Primorye costal region) and pop 15–18 in Sakhalin shared also one haplotype (H33), and pop19 (Sakhalin) and pop22 (Primorye costal region) shared one haplotype (H27), indicating gene exchange in Far Eastern Russia populations. In addition, IMa analyses indicated that an asymmetric gene flow existed between WS and VL in Far Eastern Russia (Table 3). Marine transportation seems closely related to kelp introduction from west coast of Sakhalin to Primorye costal region, because most locations are near harbors. Besides ballast water in ships likely promoted gene exchange in macroalgae such as *Undaria pinnatifida* (Harvey) Suringar [59].

Artificial cultivation enhances kelp distribution and impacts population genetic patterns [26, 54]. Gene flow reduces population differences and promotes shallow population genetic structure. Shared haplotypes H1 in China and H20 in Japan indicated that *S. japonica* in China was mainly from Hokkaido areas, confirming suggestions of Tseng et al., [25]. Pop23 in Korea shared haplotypes H27 and H29 with pop4 and pop5 in Hokkaido. Apparently, artificial cultivation facilitates the gene exchange among the populations in China, South Korea and Japan.

### Genetic diversity pattern

Ancestral Laminariales occurred along the coast of Hokkaido, which is regarded as the diversity center for kelps in the Northwestern Pacific Ocean [61]. Similar to *Undaria pinnatifida* and *Gracilaria vermiculophylla* (Ohmi) Papenfuss [30, 62], *S. japonica* exhibited the highest genetic diversity in Hokkaido. Among the 55 haplotypes analyzed with the *COI* and *trn*W-L marker systems, H1 was the most common in almost all populations (20/26), so H1 might be the ancestral haplotype. Moreover, pop7 and pop9 presented the highest genetic diversity among all populations (Fig. 2; Table 1), suggesting that the origin and diversification center may be on the Southwest coast of Hokkaido.

The invasive populations have lower genetic diversity than the source populations as reported in other introduced seaweeds, including *Gracilaria vermiculophylla* (Ohmi) Papenfuss [30], *Codium fragile* (Suringar) Hariot [63] and *Caulerpa taxifolia* (M. Vahl) C. Agardh [64]. *S. japonica* is not native to China and this kelp was firstly found in the vicinity of Dalian harbor around 1930s [25, 65], possibly because it was accidentally introduced by rafts or ships from Japan to China. It is interesting that natural populations in China have relatively high genetic diversity based on the nucleotide diversity and haplotype diversity (Table 1). We presumed that human mediated multiple introductions for breeding and cultivation contributed to high genetic diversity in China populations. Recently, selection, breeding and cultivation have reduced the genetic diversity and narrowed the genetic base to the cultivated kelp populations in China [7, 8]. In this study, the identified wild kelp populations with high genetic diversity might make important contributions to improve and enhance domesticated genepool of cultivated *S. japonica* in China.

## Conclusions

This study examined the phylogeographic architecture and population genetic diversity of *S. japonica* in its native range. The shallow phylogeographic architecture suggests a complex interaction of anthropogenic interference (marine transportation and aquaculture) and post-LGM population expansion. The coast of Hokkaido might be the origin center for *S. japonica* in the northwestern Pacific. Extensive selection and multi-generation inbreeding have already reduced genetic diversity of *S. japonica* and caused genetic degeneration of cultivars in China. The generated knowledge about the levels of diversity of wild *S. japonica* populations is an important contribution for efficient breeding and rejuvenation of cultivated *Saccharina* by improving and enhancing domesticated genepool of *S. japonica* in China.

### Availability of supporting data

The newly obtained *COI* and *trn*W-L haplotype sequences: GenBank accessions number: KT963115-KT963144 for *COI*; KT963093-KT963114 for *trn*W-L.

## Additional files

Additional file 1: Table S1. Collection details of the 26 populations of *Saccharina japonica* studied. (PDF 48 kb) Additional file 2: Figure S1. Tessellation illustration of Bayesian analysis of population structure. Each cell of the tessellation corresponds to the physical neighborhood of an observed data point. Various colors indicate genetic groupings as retrieved by phylogenetic analyses. (PDF 238 kb) Additional file 3: Table S2. Genetic differentiation (*F*_ST_) between the pairs of four genetic clusters. **,*P*< 0.001; *,*P*< 0.05 (PDF 48 kb) Additional file 4: Table S3. Pairwise *F*_ST_ estimates among *Saccharina japonica* populations. **P*< 0.05; ***P*< 0.001; ns not significant. (PDF 64 kb) Additional file 5: Figure S2. Isolation-by distance (IBD) analyses within 22 populations (except for four populations in China and Korea). Regression of genetic differentiation (estimated by *F*_ST_/(1- *F*_ST_)) against logarithm of geographical distances (km). (PDF 36 kb) Additional file 6: Figure S3. Pairwise mismatch distributions for *Saccharina japonica* inferred from mtDNA sequences. The abscissa indicates the number of pairwise differences between compared sequences. The ordinate is frequency for each value. Bar represent the observed distribution of pairwise frequencies, while the solid line shows the expected distribution. (PDF 340 kb) Additional file 7: Figure S4. Bayesian skyline plots showing effective population size as a function of time. (a) inferred from single sequence *COI*; (b) inferred from single sequence *trn*W-L;(c) inferred from combined mtDNA sequences (*COI* + *trn*W-L). The upper and lower limits of light blue trend represent the 95 % confidence intervals of HPD analysis. (PDF 150 kb) Additional file 8: Figure S5. Maximum clade credibility coalescent tree, based on *COI*. (PDF 57 kb)
