# Supplementary material for: Phylogeographic data revealed shallow genetic structure in the kelp Saccharina japonica (Laminariales, Phaeophyta)
Source: BMC Evol Biol. 2015 Nov 2;15:237. doi: 10.1186/s12862-015-0517-8 (PMC4630829; doi:10.1186/s12862-015-0517-8)
Supplement: Additional file 1: Table S1. — Collection details of the 26 populations of Saccharina japonica studied. (PDF 48 kb) [file 12862_2015_517_MOESM1_ESM.pdf]

Table S1 Collection details of the 26 populations of *Saccharina japonica* studied.

Population number	Sample site	Sample size	Coordinates	Data of collection
**1**	Hokkaido, Japan	27	N45°27′ E141°38′	2012.12
**2**	Hokkaido, Japan	6	N45°24 ′E141°40′	2012.12
**3**	Hokkaido, Japan	22	N43°20′ E140°23′	2012.07
**4**	Hokkaido, Japan	10	N43°11′ E140°47′	2012.07
**5**	Hokkaido, Japan	24	N43°12′ E140°51′	2012.07
**6**	Hokkaido, Japan	21	N43°12′ E141°00′	2012.07
**7**	Hokkaido, Japan	28	N41°46′ E140°41′	2011.10
**8**	Hokkaido, Japan	26	N42°06′ E140°35′	2011.10
**9**	Hokkaido, Japan	7	N42°31′ E140°23′	2012.07
**10**	Hokkaido, Japan	28	N43°17′ E145°31′	2012.07
**11**	Hokkaido, Japan	32	N43°58′ E144°52′	2012.06
**12**	Kunashir Island, Russia	28	N44°03′ E145°43′	2011.09
**13**	Sakhalin, Russia	24	N46°06′ E143°18′	2011.09
**14**	Sakhalin, Russia	29	N46°36′ E142°54′	2011.09
**15**	Sakhalin, Russia	28	N46°25′ E141°51′	2013.09
**16**	Sakhalin, Russia	21	N46°32′ E141°48′	2011.09
**17**	Sakhalin, Russia	30	N46°32′ E141°48′	2011.09
**18**	Sakhalin, Russia	16	N47°08′ E142°03′	2011.09
**19**	Sakhalin, Russia	26	N48°50′ E141°55′	2011.09
**20**	Sakhalin, Russia	29	N48°50′ E141°55′	2011.09
**21**	Primorsky, Russia	30	N43°57′ E135°27′	2012.10
**22**	Primorsky, Russia	6	N43°06′E131°52′	2011.09
**23**	South Korea	27	N37°47′ E128°55′	2013.06
**24**	Liaoning, China	27	N39°05′ E 122°47′	2014.07
**25**	Shandong, China	30	N37°29′ E121°34′	2011.05
**26**	Shandong, China	30	N37°09′ E122°35′	2011.05
