# Supplementary material for: Phylogeographic data revealed shallow genetic structure in the kelp Saccharina japonica (Laminariales, Phaeophyta)
Source: BMC Evol Biol. 2015 Nov 2;15:237. doi: 10.1186/s12862-015-0517-8 (PMC4630829; doi:10.1186/s12862-015-0517-8)

Figure S1 Tessellation illustration of Bayesian analysis of population structure. Each cell of the tessellation corresponds to the physical neighborhood of an observed data point. Various colors indicate genetic groupings as retrieved by phylogenetic analyses.

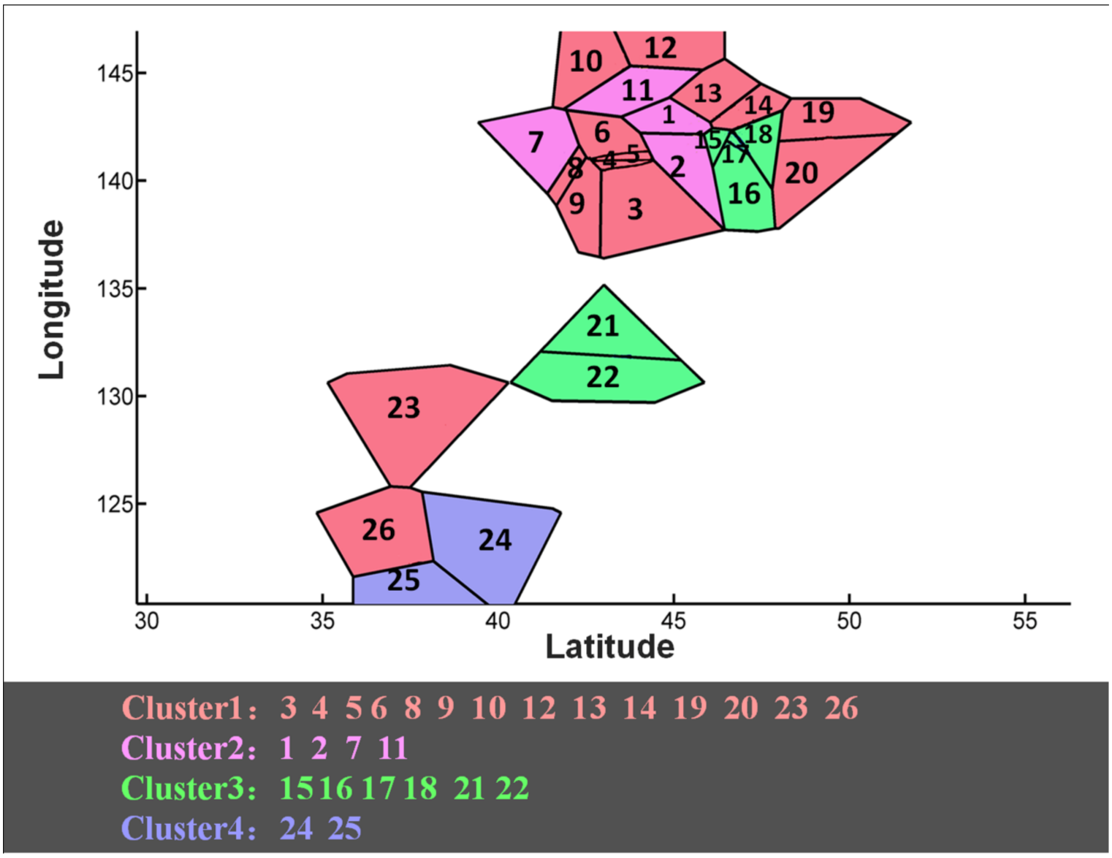

Supplement: Additional file 2: Figure S1. — Tessellation illustration of Bayesian analysis of population structure. Each cell of the tessellation corresponds to the physical neighborhood of an observed data point. Various colors indicate genetic groupings as retrieved by phylogenetic analyses. (PDF 238 kb) [file 12862_2015_517_MOESM2_ESM.pdf]
