# Supplementary material for: Phylogeographic data revealed shallow genetic structure in the kelp Saccharina japonica (Laminariales, Phaeophyta)
Source: BMC Evol Biol. 2015 Nov 2;15:237. doi: 10.1186/s12862-015-0517-8 (PMC4630829; doi:10.1186/s12862-015-0517-8)
Supplement: Additional file 3: Table S2. — Genetic differentiation (F ST) between the pairs of four genetic clusters. **,P< 0.001; *,P< 0.05 (PDF 48 kb) [file 12862_2015_517_MOESM3_ESM.pdf]

Table S2 Genetic differentiation (F_{ST}) between the pairs of four genetic clusters

	**Cluster 1**	**Cluster 2**	**Cluster 3**	**Cluster 4**
**Cluster 1**				
**Cluster 2**	0.22309**			
**Cluster 3**	0.62105**	0.46619**		
**Cluster 4**	0.77709**	0.56143**	0.87016**	

**, *P* < 0.001; *, *P* < 0.05
