# Supplementary material for: Phylogeographic data revealed shallow genetic structure in the kelp Saccharina japonica (Laminariales, Phaeophyta)
Source: BMC Evol Biol. 2015 Nov 2;15:237. doi: 10.1186/s12862-015-0517-8 (PMC4630829; doi:10.1186/s12862-015-0517-8)
Supplement: Additional file 4: Table S3. — Pairwise F ST estimates among Saccharina japonica populations. *P< 0.05; **P< 0.001; ns not significant. (PDF 64 kb) [file 12862_2015_517_MOESM4_ESM.pdf]

Table S3 Pairwise F_{ST} estimates among *Saccharina japonica* populations. * P < 0.05; ** P < 0.001; ns, not significant

	1	2	3	4	5	6	7	8	9	10	11	12	13	14	15	16	17	18	19	20	21	22	23	24	25
1	0																								
2	0.1161^{ns}	0																							
3	0.6753**	0.9147**	0																						
4	0.5431**	0.6304**	0.2321^{ns}	0																					
5	0.5431**	0.5984**	0.2503*	0.1898^{ns}	0																				
6	0.6456**	0.8568**	0.0023^{ns}	0.1759^{ns}	0.2286^{ns}	0																			
7	0.4305**	0.3840^{ns}	0.2891**	0.2164*	0.2713**	0.2791*	0																		
8	0.5940**	0.7238**	0.1189^{ns}	0.1499*	0.2281**	0.0998*	0.2935**	0																	
9	0.5847**	0.8292**	0.0000^{ns}	0.0805^{ns}	0.1482^{ns}	-0.0573^{ns}	0.1912*	0.0397^{ns}	0																
10	0.7019**	0.9297**	0.0000^{ns}	0.2765^{ns}	0.2807*	0.0141^{ns}	0.3185**	0.1401*	0.0000^{ns}	0															
11	0.4879**	0.6812**	0.1298^{ns}	0.1801*	0.2528**	0.1106*	0.3199**	0.1481**	0.0556^{ns}	0.1494*	0														
12	0.6780**	0.8843**	-0.0089^{ns}	0.2209^{ns}	0.2621*	0.0018^{ns}	0.3133**	0.1198*	-0.0614^{ns}	0.0000^{ns}	0.1285*	0													
13	0.6375**	0.8245**	-0.0037^{ns}	0.1598^{ns}	0.2283**	-0.0016^{ns}	0.2813**	0.0954*	-0.0595^{ns}	0.0066^{ns}	0.1048*	0.0025^{ns}	0												
14	0.6413**	0.8124**	0.0114^{ns}	0.1643*	0.2391*	0.0110^{ns}	0.3089**	0.1022*	-0.0449^{ns}	0.0222^{ns}	0.1108*	0.0170^{ns}	0.0124^{ns}	0											
15	0.6556**	0.8428**	-0.0089^{ns}	0.1816^{ns}	0.2457*	-0.0024^{ns}	0.3082**	0.1046*	-0.0614^{ns}	0.0000^{ns}	0.1129*	0.0000^{ns}	0.0005^{ns}	0.0138^{ns}	0										
16	0.6227**	0.8077**	0.0023^{ns}	0.1426^{ns}	0.2145*	0.0000^{ns}	0.2746**	0.0885*	-0.0573^{ns}	0.0141^{ns}	0.0990*	0.0059^{ns}	0.0004^{ns}	0.0120^{ns}	0.0018^{ns}	0									
17	0.6901**	0.8932**	-0.0116^{ns}	0.2384^{ns}	0.2753*	0.0031^{ns}	0.3266^{ns}	0.1279*	-0.0624^{ns}	-0.0034^{ns}	0.1358*	0.0002^{ns}	0.0045^{ns}	0.0196^{ns}	-0.0218^{ns}	0.0085^{ns}	0								
18	0.6180**	0.8275**	0.0206^{ns}	0.1395^{ns}	0.2017^{ns}	0.0021^{ns}	0.2510**	0.0844^{ns}	-0.0503^{ns}	0.0370^{ns}	0.0972^{ns}	0.0081$^{ns\ v}$	-0.0030^{ns}	0.0077^{ns}	-0.0018^{ns}	-0.0031^{ns}	0.0109^{ns}	0							
19	0.6694**	0.8776**	-0.0066^{ns}	0.2086^{ns}	0.2529*	0.0010^{ns}	0.3040**	0.1143^{ns}	-0.0606^{ns}	0.0029^{ns}	0.1235*	0.0001$^{ns\ *}$	0.0012^{ns}	0.0152^{ns}	-0.0008^{ns}	0.0042^{ns}	0.0006^{ns}	0.0062^{ns}	0						
20	0.6002**	0.7430**	-0.0099^{ns}	0.1157^{ns}	0.2104*	-0.0070^{ns}	0.2983**	0.0769*	-0.0618^{ns}	-0.0012^{ns}	0.0843*	-0.0008**	-0.0025^{ns}	0.0089^{ns}	-0.0005^{ns}	-0.0037^{ns}	0.0016^{ns}	-0.0119^{ns}	-0.0025^{ns}	0					
21	0.7098**	0.9336**	0.0000^{ns}	0.2899^{ns}	0.2901*	0.0175^{ns}	0.3276^{ns}	0.1466*	0.0000^{ns}	0.0000^{ns}	0.1554*	0.0025^{ns}	0.0095^{ns}	0.0254^{ns}	0.0025^{ns}	0.0175^{ns}	-0.0011^{ns}	0.0419^{ns}	0.0056^{ns}	0.0012^{ns}	0				
22	0.5646**	0.8000^{ns}	0.0000^{ns}	0.0400^{ns}	0.1197^{ns}	-0.0839^{ns}	0.1645**	0.0127^{ns}	0.0000^{ns}	0.0000^{ns}	0.0299^{ns}	-0.0870^{ns}	-0.0856^{ns}	-0.0705^{ns}	-0.0870^{ns}	-0.0839^{ns}	-0.0877^{ns}	-0.0787^{ns}	-0.0864^{ns}	-0.0873^{ns}	0.0000^{ns}	0			
23	0.5633**	0.6738**	0.0382^{ns}	0.0627^{ns}	0.1593*	0.0335^{ns}	0.2881**	0.0889*	-0.0233^{ns}	0.0516^{ns}	0.0990*	0.0460^{ns}	0.0359^{ns}	0.0455^{ns}	0.0415^{ns}	0.0317^{ns}	0.0509^{ns}	0.0231^{ns}	0.0426^{ns}	0.0330^{ns}	0.0555^{ns}	-0.0492^{ns}	0		
24	0.8462**	0.9607**	1.0000**	0.8538**	0.7404**	0.9603**	0.5304**	0.8402**	1.0000**	1.0000**	0.8575**	0.9649**	0.9275**	0.9054**	0.9322**	0.9236**	0.9666**	0.9563**	0.9637**	0.8483**	1.0000**	1.0000**	0.7893**	0	
25	0.8534**	0.9641**	1.0000**	0.8639**	0.7519**	0.9626**	0.5449**	0.8478**	1.0000**	1.0000**	0.8637**	0.9667**	0.9314**	0.9100**	0.9356**	0.9280**	0.9682**	0.9592**	0.9656**	0.8552**	1.0000**	1.0000**	0.7983**	0.0000^{ns}	0
26	0.6496**	0.8222**	0.0505^{ns}	0.1825*	0.2502**	0.0401^{ns}	0.3153**	0.1182*	-0.0127^{ns}	0.0647^{ns}	0.1278*	0.0491^{ns}	0.0370^{ns}	0.0461^{ns}	0.0398^{ns}	0.0356^{ns}	0.0530^{ns}	0.0349^{ns}	0.0465^{ns}	0.0258^{ns}	0.0690^{ns}	-0.0385^{ns}	0.0586^{ns}	0.8915**	0.8966**
