# Supplementary material for: Phylogeographic data revealed shallow genetic structure in the kelp Saccharina japonica (Laminariales, Phaeophyta)
Source: BMC Evol Biol. 2015 Nov 2;15:237. doi: 10.1186/s12862-015-0517-8 (PMC4630829; doi:10.1186/s12862-015-0517-8)

Figure S2 Isolation-by distance (IBD) analyses within 22 populations (except for four populations in China and Korea). Regression of genetic differentiation (estimated by $F_{ST}/(1- F_{ST})$) against logarithm of geographical distances (km).

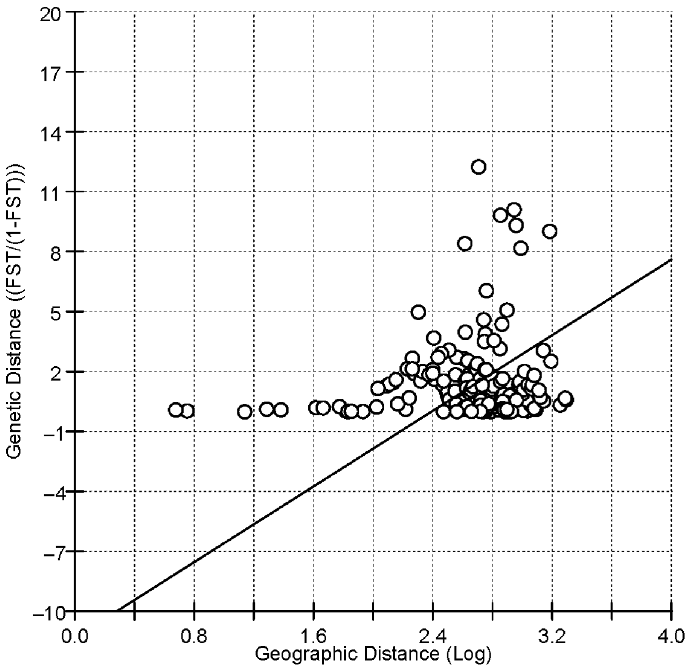

Supplement: Additional file 5: Figure S2. — Isolation-by distance (IBD) analyses within 22 populations (except for four populations in China and Korea). Regression of genetic differentiation (estimated by F ST/(1- F ST)) against logarithm of geographical distances (km). (PDF 36 kb) [file 12862_2015_517_MOESM5_ESM.pdf]
