# Supplementary material for: Phylogeographic data revealed shallow genetic structure in the kelp Saccharina japonica (Laminariales, Phaeophyta)
Source: BMC Evol Biol. 2015 Nov 2;15:237. doi: 10.1186/s12862-015-0517-8 (PMC4630829; doi:10.1186/s12862-015-0517-8)

Figure S3 Pairwise mismatch distributions for *Saccharina japonica* inferred from mtDNA sequences. The abscissa indicates the number of pairwise differences between compared sequences. The ordinate is frequency for each value. Bar represent the observed distribution of pairwise frequencies, while the solid line shows the expected distribution.

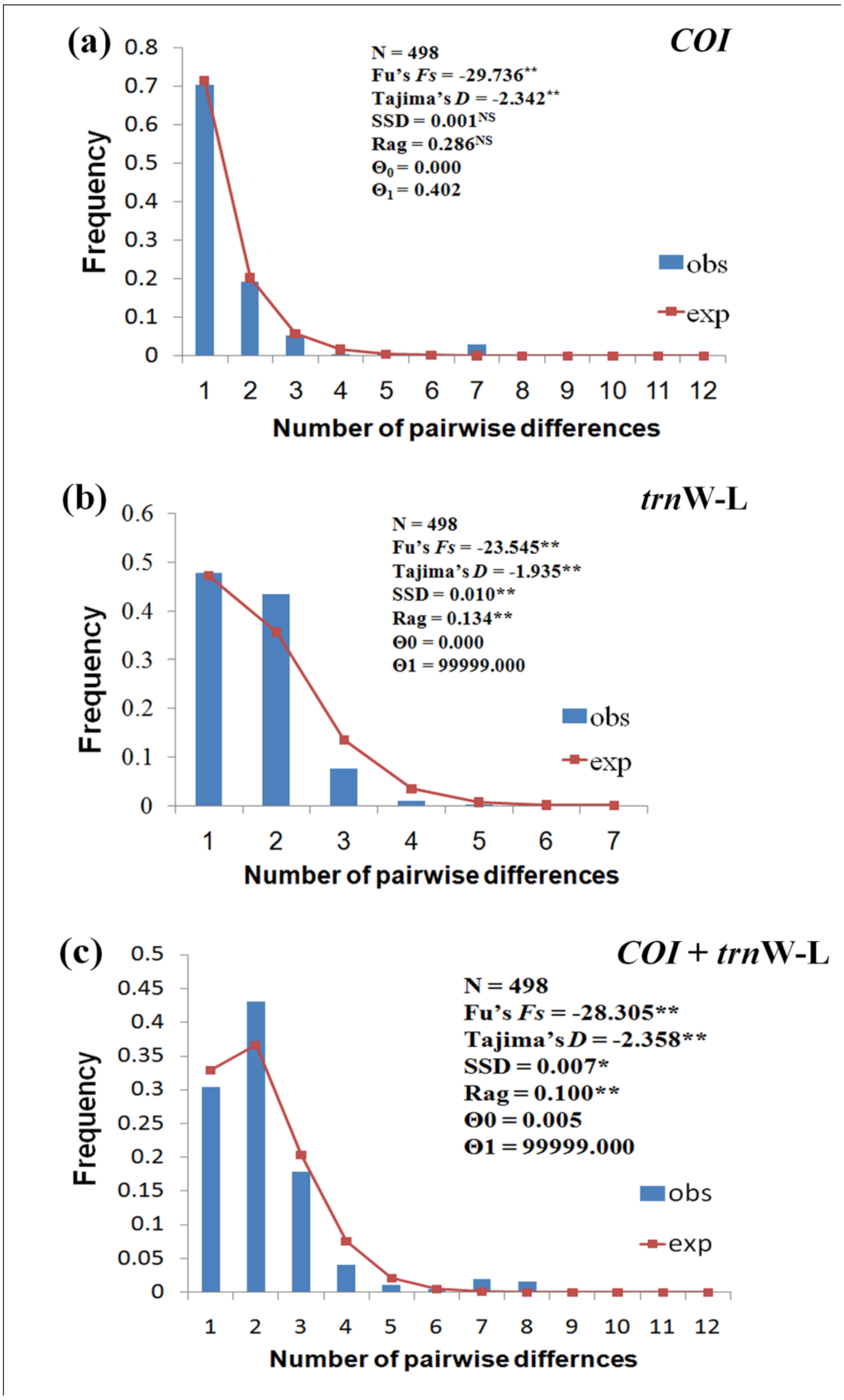

Supplement: Additional file 6: Figure S3. — Pairwise mismatch distributions for Saccharina japonica inferred from mtDNA sequences. The abscissa indicates the number of pairwise differences between compared sequences. The ordinate is frequency for each value. Bar represent the observed distribution of pairwise frequencies, while the solid line shows the expected distribution. (PDF 340 kb) [file 12862_2015_517_MOESM6_ESM.pdf]
