# Supplementary material for: Phylogeographic data revealed shallow genetic structure in the kelp Saccharina japonica (Laminariales, Phaeophyta)
Source: BMC Evol Biol. 2015 Nov 2;15:237. doi: 10.1186/s12862-015-0517-8 (PMC4630829; doi:10.1186/s12862-015-0517-8)

Figure S4 Bayesian skyline plots showing effective population size as a function of time. (a) inferred from single sequence *COI*; (b) inferred from single sequence *trn*W-L;(c) inferred from combined mtDNA sequences (*COI*+*trn*W-L). The upper and lower limits of light blue trend represent the 95% confidence intervals of HPD analysis.

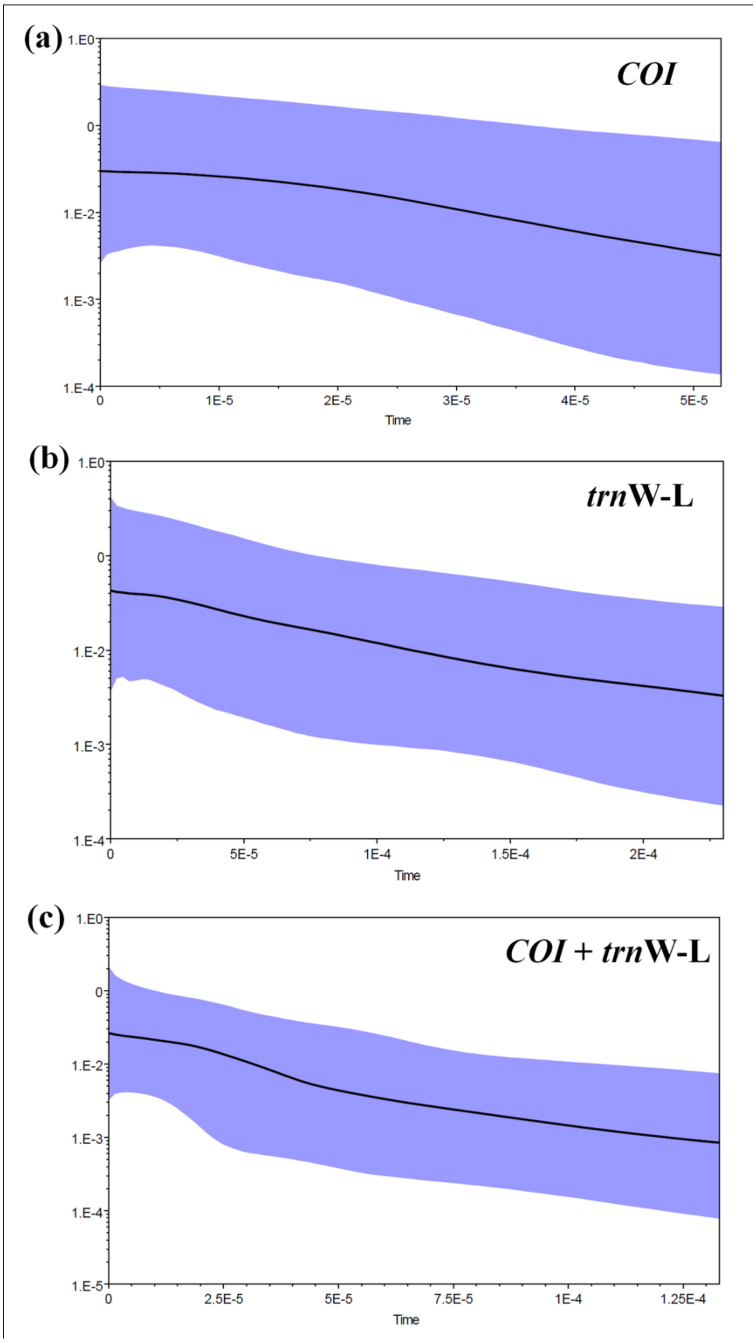

Supplement: Additional file 7: Figure S4. — Bayesian skyline plots showing effective population size as a function of time. (a) inferred from single sequence COI; (b) inferred from single sequence trnW-L;(c) inferred from combined mtDNA sequences (COI + trnW-L). The upper and lower limits of light blue trend represent the 95 % confidence intervals of HPD analysis. (PDF 150 kb) [file 12862_2015_517_MOESM7_ESM.pdf]
