# Supplementary figures and images for: Phylogeographic data revealed shallow genetic structure in the kelp Saccharina japonica (Laminariales, Phaeophyta)
Source: BMC Evol Biol. 2015 Nov 2;15:237. doi: 10.1186/s12862-015-0517-8 (PMC4630829; doi:10.1186/s12862-015-0517-8)

Figure S5 Maximum clade credibility coalescent tree based on *COI*.

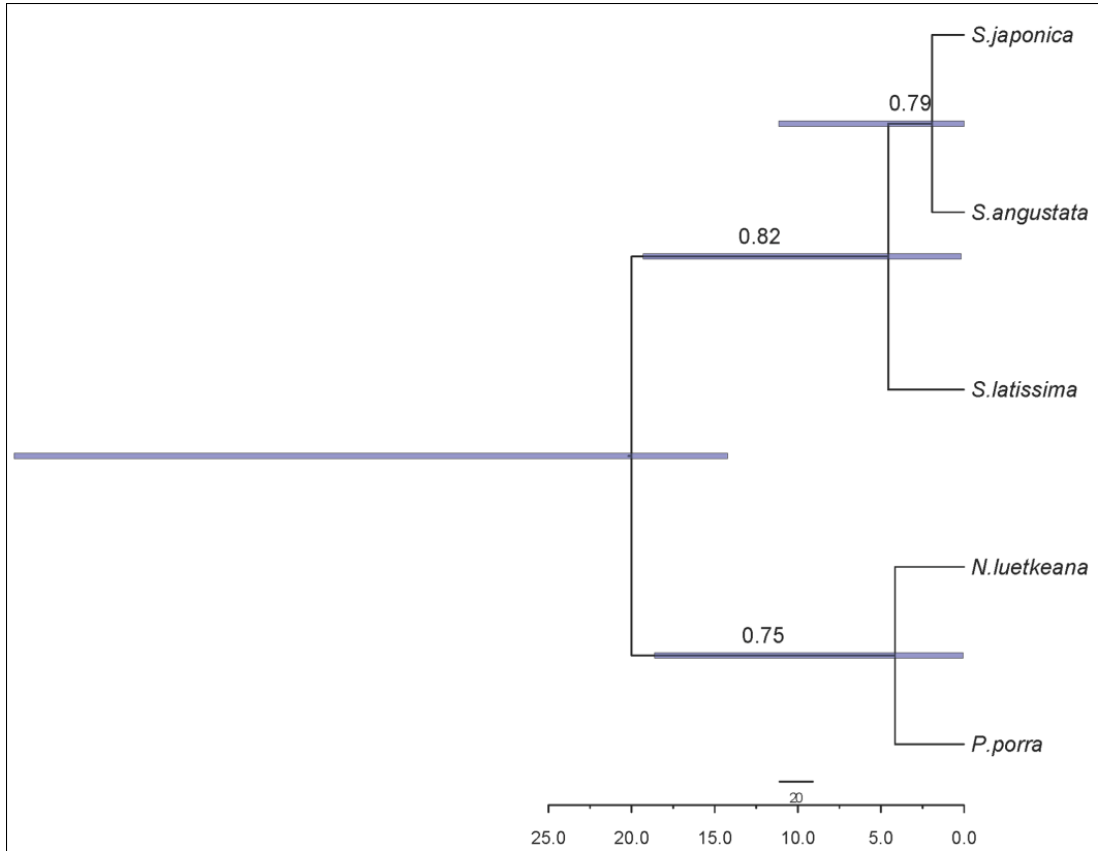

Supplement: Additional file 8: Figure S5. — Maximum clade credibility coalescent tree, based on COI. (PDF 57 kb) [file 12862_2015_517_MOESM8_ESM.pdf]
